# From Living Room to Operating Room for Patients with Pulmonary Surgeries: A Systematic Review of Literature

**DOI:** 10.3390/jcm14238605

**Published:** 2025-12-04

**Authors:** Fatma Öztürk, Zeynep İclal Sağ, Ömer Bayrak, Esra Pehlivan

**Affiliations:** 1 Physiotherapy Program, Vocational School, Medipol University, Istanbul 34815, Turkey; fatma.ozturk@medipol.edu.tr; 2 Department of Physiotherapy and Rehabilitation, Faculty of Health Sciences, Yalova University, Yalova 77200, Turkey; zeynep.sag@yalova.edu.tr; 3 Department of Physiotherapy and Rehabilitation, Faculty of Health Sciences, Haliç University, Istanbul 34060, Turkey; omerbayrak@halic.edu.tr; 4 Department of Physiotherapy and Rehabilitation, Faculty of Hamidiye Health Sciences, University of Health Sciences, Selimiye Mah. Tıbbiye Cad. No: 38, Üsküdar, Istanbul 34668, Turkey

**Keywords:** home-based exercises, prehabilitation, pulmonary surgery, lung function, exercise capacity, quality of life

## Abstract

**Introduction:** This systematic review aimed to evaluate the effectiveness of preoperative home-based exercise training on clinical outcomes in patients undergoing pulmonary surgery. **Methods:** A comprehensive literature search was conducted on 25 October 2024, in PubMed, Scopus, PEDro, and Cochrane databases for English-language studies published between 2014 and 2024. Randomized controlled trials, non-randomized trials, feasibility studies, and cohort studies involving adults scheduled for pulmonary surgery and receiving home-based prehabilitation were included. Methodological quality was assessed using the PEDro Quality Assessment scale and i-CONTENT tool. **Results:** Six studies involving patients undergoing pulmonary surgery were included, of which three were randomized controlled trials. Two studies were rated as excellent quality, one as good, two as moderate, and one as poor. Interventions ranged from 2 to 12 weeks and generally included aerobic or functional training. Compared to usual care, participants in home-based exercise groups showed improvements in physical capacity (e.g., 6-min walk test, sit-to-stand), enhanced quality of life, and reduced hospital length of stay. **Conclusions:** Preoperative home-based exercise training appears to be a feasible and effective approach to improve functional outcomes and reduce hospitalization after pulmonary surgery. However, heterogeneity in study design and quality limits generalizability, highlighting the need for further high-quality trials.

## 1. Introduction

Pulmonary surgeries are one of the most frequently used methods in the treatment of respiratory system diseases. However, these surgeries carry various complication risks such as decreased respiratory function in the postoperative period, respiratory tract infections and prolonged hospitalization [1,2]. Postoperative complications can impede the patient’s recovery process, leading to longer hospital stays and additional healthcare costs, thereby placing an extra burden on the healthcare system [3]. In addition, these complications may significantly reduce patients’ health-related quality of life (QoL) by causing persistent fatigue, pain, dyspnea, and limitations in daily functioning after surgery [4].

Decreased functional capacity is a common outcome in the postoperative period due to systemic inflammation and surgical stress. Many patients have difficulty returning to their initial functional level after surgery. In particular, reduced functional capacity has been recognized as an important determinant of both morbidity and mortality after lung surgery [5]. Additionally, even though QoL usually improves within 3 to 6 months after surgery, a significant proportion of patients face long-term functional limitations, with symptoms such as fatigue, pain and shortness of breath persisting for up to 1 to 2 years [6].

In order to overcome the complications and increase the patient’s ability to tolerate surgical stress, the concept of “prehabilitation” has emerged as an approach focused on increasing preoperative physiological reserves [7]. Although the effectiveness of prehabilitation has been demonstrated, it is often limited by major barriers such as difficulties in participant recruitment, high attrition rates, low adherence—where the sustainability of exercise sessions is reduced due to factors such as low motivation, limited accessibility, or progression of the disease—small sample sizes, insurance constraints, and high costs [8,9]. In this context, home-based prehabilitation exercise programs offer a valuable option for individuals facing challenges related to accessibility, transportation barriers, and high costs, as it can be easily integrated into patients’ daily routines and implemented in their home environment. These programs not only allow patients to exercise without the need for hospital visits, but they also increase cost-effectiveness and facilitate better patient compliance, particularly by enhancing adherence to the prescribed home-based exercise sessions, recommended training frequency, and overall continuity of the program [10]. In addition, both exercise capacity and respiratory function can be improved through prehabilitation programs in patients preparing to undergo pulmonary surgery. These improvements can help reduce the risk of postoperative pulmonary complications and shorten hospital length of stay (LOS), leading to a better recovery process and improved postoperative outcomes [11,12].

There are studies in the literature examining the effectiveness of preoperative exercise programs [5,13]; however, to the best of our knowledge, no systematic review specifically addressing the effects of home-based prehabilitation programs in pulmonary surgeries is available. Therefore, the aim of this systematic review is to examine the efficacy of home-based prehabilitation programs on postoperative outcomes like functional capacity, LOS, respiratory function, physical performance, QoL, frailty and complications in pulmonary surgery patients.

## 2. Material and Methods

To ensure methodological transparency and standards, our systematic review was pre-registered with International Prospective Register of Systematic Reviews (PROSPERO) (ID: CRD42024606245). This review was conducted in accordance with Cochrane guidelines and the Preferred Reporting Items for Systematic Reviews and Meta-Analyses (PRISMA) guidelines. A completed PRISMA 2020 checklist has been provided as Supplementary Materials.

Trials were identified from four databases: PubMed, Scopus, PEDro and Cochrane. A comprehensive search strategy was developed using three main keyword categories: (1) pulmonary surgery–related terms, (2) home-based or unsupervised exercise–related terms and (3) preoperative or prehabilitation-related terms. Each category included multiple synonyms combined with the Boolean operator OR, and the three categories were subsequently combined using AND. The databases were searched on 25/10/2024 using these predefined keyword blocks and their variations to ensure a thorough and systematic identification of relevant studies. The full list of search terms and keyword combinations used in this review is provided in Appendix A. We included randomized controlled trials (RCTs), non-RCTs, controlled clinical trials, feasibility studies, pilot studies and retrospective cohort studies published in English between 2014 and 2024.

### 2.1. Search Strategy

Duplicate articles were removed from the articles found as a result of the search and the remaining studies were evaluated for eligibility according to their abstracts. Studies to be included in this systematic review were selected by reading their full texts according to the specified inclusion and exclusion criteria. Inclusion criteria were determined as; conducted within the last 10 years, having accessible full text, including adults who underwent pulmonary surgery, having applied any home-based exercise training intervention, and having an outcome measure evaluating postoperative recovery parameters (such as respiratory function, functional capacity, QoL, pain, dyspnea, fatigue and depression).

Exclusion criteria were defined as study language other than English, no abstract, wrong publication type (clinical trial record, letter to the editor, protocol, etc.), wrong population, wrong study design, wrong intervention and studies without a control group.

The Rayyan AI-powered tool (Rayyan Systems Inc., Cambridge, MA, USA) was used to export the studies for detailed selection via the Endnote 20 (Clarivate Analytics, Philadelphia, PA, USA) [14].

### 2.2. Quality Assessment Process and Bias Analysis

To avoid bias, only the inclusion criteria were considered in these scans and statistical results were ignored. The selection and assessment of these studies from the literature was carried out separately by F.B. and Z.İ.S. The differences between the included studies were discussed with a third researcher, Ö.B., and a joint decision was reached.

The RCTs included in the systematic review were assessed using the PedRO Quality Assessment tool. This tool consists of 11 items to measure the methodological quality of RCTs, with each item being scored as “yes” or “no”; the total score ranges from 0 to 10. Higher scores indicate greater methodological strength. Studies scoring 9–10 on the PEDro scale are of excellent quality, meeting all critical methodological criteria. Scores of 6–8 indicate good quality and meet most important standards. Scores of 4–5 represent moderate quality and contain some deficiencies. Scores of 0–3 indicate poor quality and the reliability of the findings is limited [15].

The Consensus on Therapeutic Exercise and Training (i-CONTENT) tool was developed through a structured expert consensus process using Delphi methodology to ensure content validity. Designed to assess the scientific validity and standards of practice of therapeutic exercise and training programs, the tool provides guidance for the systematic evaluation of the design and implementation of exercise interventions in both clinical and research settings. It evaluates key components such as rationale, dosing, specificity, safety, individualization and feasibility [16]. Previous studies have reported acceptable inter-rater agreement, and due to its systematic structure and increasing use in exercise-based systematic reviews, the i-CONTENT tool is considered a scientifically robust and appropriate instrument for evaluating the content of therapeutic exercise programs [17,18]. The included studies were analyzed using the i-CONTENT tool.

Data are presented with descriptive statistics and results are categorized by study type, sample characteristics and exercise intervention. Publication bias is addressed with qualitative interpretation. Heterogeneity analysis is examined without meta-analysis and consistency/differences between studies are discussed.

## 3. Results

**Figure 1:**
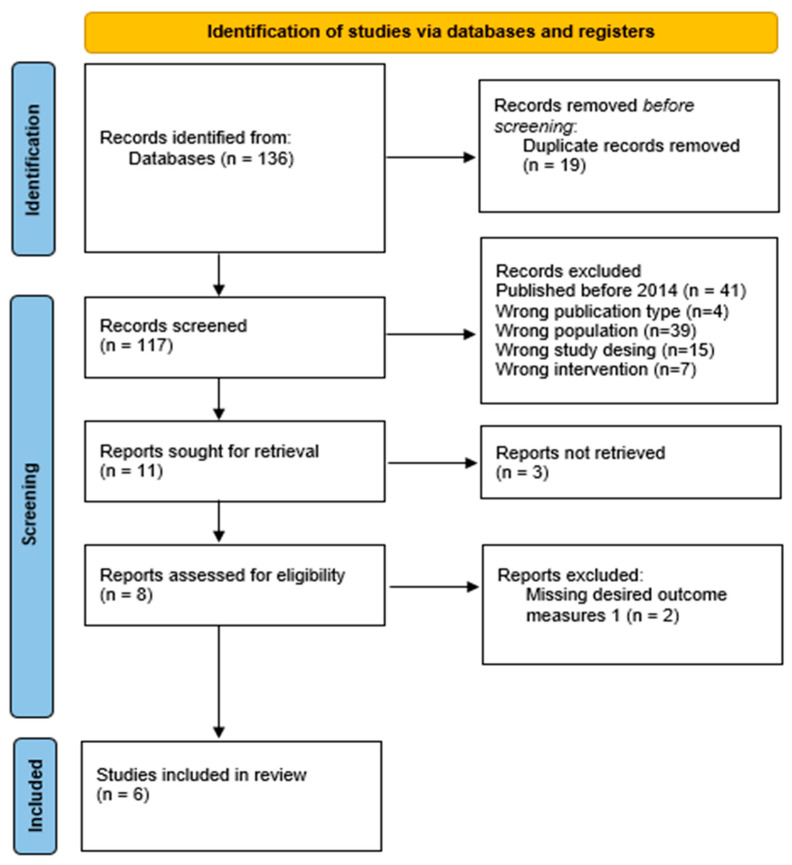
Flow Chart of Study (right here).

A total of 136 trials were identified as a result of the searches. Of these, 19 were found to be duplicates. After reviewing the abstracts of the remaining 117 studies, 6 were found to meet the inclusion criteria. (Figure 1)

Three of the six included studies were RCTs, two were retrospective cohort studies and one was a pilot study. The included trials were conducted in different countries, including China, Canada, Portugal and Japan. The total number of participants included in the preoperative evaluation trials was 527. These participants were mostly lung cancer patients and lung transplant candidates. The age and gender distributions of the intervention and control groups in trials that provided important evidence on the effectiveness of home-based pulmonary rehabilitation are shown in Table 1.

The methodological quality of the six studies included in this systematic review was assessed using the PedRO quality assessment scale. Of the studies, Machado (2024) and Patel (2023) were of high methodological quality, both scoring 9/10. These studies met important criteria such as randomization, allocation concealment, assessor blinding, and adherence to pre-intention-to-treat analysis. While the study by Liu (2020) has a good quality score of 7/10, Saito (2021) and Singer (2018) are in the medium quality category with a score of 5/10, losing points mainly due to the lack of blinding and randomization. The study by Massierer (2020) was rated as medium quality with a score of 4/10. Overall, the studies differ in methodological quality, with higher-quality studies yielding more reliable findings (Table 2).

All the trials used home-based rehabilitation programs before pulmonary surgery. These programs included respiratory exercises, chest physiotherapy, aerobic exercises, strength exercises and personalized exercises, and in some trials [19,22] applications that have become popular with the development of technology. Participants were supported by telephone advice throughout the interventions. The control groups received a usual care program. The interventions were delivered between 2 weeks and 12 weeks before lung surgery (Table 3).

The scientific validity and implementation standards of the interventions were assessed with the i-CONTENT tool. Based on the evaluations, the reviewed studies were generally of high quality and offer robust evidence regarding the feasibility, safety, and effectiveness of the exercise programs. All phases of the exercise programs in the studies, from patient selection to dosage, type and trainer support, were carefully planned and designed to meet the clinical needs of the patients. The types of exercise were defined as aerobic exercises to increase cardiovascular endurance and resistance exercises to strengthen muscles, and each component of the program was designed to meet the needs of the patients. In these studies, the safety of the program was ensured by well-documented and widely used current rehabilitation approaches. In addition, the use of trained instructors and mobile health technologies increased participants’ adherence to the exercises and ensured that they were exercising safely, while also allowing changes in the studied parameters to be tracked. (Table 4).

However, the limited trainer support in the studies, especially the remote monitoring of the exercises and the lack of direct interaction with the trainer, posed a moderate risk. However, this was generally compensated for by mobile applications and telephone support. The outcome measures in the studies were carried out using valid and reliable instruments and accurately assessed the effectiveness of the exercises. Overall, the studies showed strong performance in terms of exercise program delivery processes, patient compliance and safety, providing solid data for future larger trials.

The evaluation methods in the interventions included factors such as QoL, physical performance [handgrip strength (HGS), 6-min walk test (6MWT), incremental shuttle walk test (ISWT), 5 times sit to stand test (5STS)], exercise adherence, safety and LOS. These data were collected at baseline, after surgery and again after a period of time (usually between 3 and 12 weeks).

### 3.1. Physical Performance

In the study by Li et al., the prehabilitation group showed an increase in 6MWT both in the preoperative period (mean 45.1 m) and on the 30th day after surgery (mean 21.5 m), whereas the control group showed a decrease in physical performance. The prehabilitation group achieved a mean increase in 6MWT of 60.9 m from baseline to the day of surgery compared to the control group (95% CI: 32.4–89.5; *p* < 0.001). The prehabilitation group also achieved a significant increase in forced vital capacity (FVC) compared to the control group (mean difference: 0.35 L; 95% CI: 0.05–0.66; *p* = 0.021) [7]. In the study by Patel et al., the move for surgery group increased in EQ-5D-5L™ mobility dimensions (*p* = 0.008) and daily step counts compared to baseline [19].

In the study by Machado et al., the preoperative home-based exercise training group walked 147.4 m further than the control group after surgery (95% CI: 17.3–264.2; *p* = 0.027), while the preoperative home-based exercise training group showed faster results in the 5STS test than before surgery (mean difference: −1.8 s; 95% *CI*: −0.1 to −3.7; *p* = 0.041). There was no difference in HGS (*p* > 0.05) [20].

In the retrospective study by Massierer et al., 54.7% of patients participating in the home-based prehabilitation program were able to increase or maintain their 6MWT, while only 45.3% demonstrated a significant decline. The group showing improvement achieved an average increase of +85.8 m, whereas the group with no change maintained a largely stable 6MWT. Additionally, a moderate positive correlation was observed between the final pre-transplant 6MWT and the post-transplant 6MWT (*r* = 0.528; *p* < 0.001) [8]. These findings suggest that home-based programs may help preserve or enhance functional capacity in a substantial proportion of patients, even during prolonged pre-transplant waiting periods.

Singer et al. observed improvement in frailty scores of participants after a home-based pulmonary rehabilitation program with the help of a mobile application. Short Physical Performance Battery (SPPB) scores improved in 54% (7/13), and frailty was resolved in 3 of 5 participants who were frail at baseline with ≤9 points (from 9.7 ± 1.1 to 10.8 ± 1.5, *p* = 0.08). Similarly, Fried Frailty Phenotype (FFP) scores improved in 62%, and frailty was resolved in 4 of 6 participants who were frail at baseline (from 2.4 ± 1.1 to 1.7 ± 1.4, *p* = 0.07). However, in this study conducted with 15 participants with a mean age of 63 years, no overall improvement was found in 6MWT, grip strength, Low-Intensity Physical Activity (LT-VLA) and Duke Activity Status Index (DASI) values (*p* ≥ 0.26) [22].

### 3.2. Quality of Life

In the study by Patel et al. using the Fitbit^®^ device, the overall health score in the Move For Surgery group increased from 69.38 ± 17.11 to 79.60 ± 11.63 (*p* < 0.001). There was no significant change in the control group. There were also significant improvements in mobility (*p* = 0.008) and pain/discomfort (*p* < 0.001) in the move for surgery group [19].

In the study by Machado et al., the preoperative home-based exercise training group had significantly and clinically better global QoL scores than the control group both before surgery (mean difference: 13.5 points; 95% CI: 2.4–24.6; *p* = 0.019) and one month after surgery (mean difference: 12.4 points; 95% CI: 1.3–23.4; *p* = 0.029). However, pain (*p* = 0.041) and loss of appetite (*p* = 0.024) were also less common in the preoperative home-based exercise training group one month after surgery [20].

### 3.3. Length of Hospital Stay

In the study by Patel et al., the rate of hospital stays longer than five days was 7% in the move for surgery group, compared with 24% in the control group (*p* = 0.021). The mean LOS was 2.67 ± 1.61 days in the move for surgery group and 4.44 ± 3.48 days in the control group (*p* = 0.002) [19]. Another study found no significant difference in LOS and intercostal catheterization time between the groups [21].

### 3.4. Postoperative Complications

In the study by Li et al., four patients in the prehabilitation group experienced Clavien-Dindo class II complications (e.g., atrial fibrillation, sinus tachycardia, wound infection); two patients in the control group experienced class II complications and three patients experienced class III complications (e.g., active bleeding, pneumonia, acute renal failure). There was no difference in Quality of recovery-9 (QoR-9)scores between groups in the first 3 days after surgery [7].

In the study by Saito et al., the home-based preoperative pulmonary rehabilitation group had a lower incidence of postoperative complications (*p* = 0.04). Clavien-Dindo Class I: The home-based preoperative pulmonary rehabilitation group also had a significantly lower rate of postoperative complications (*RR*: 0.55, 95% *CI*: 0.30–1.02; *p* = 0.05) [21].

## 4. Discussion

This review evaluated the effects of preoperative home-based exercise training programs for patients undergoing pulmonary surgery. The results showed that home exercise programs before pulmonary surgery were effective in improving physical performance and QoL. Significant improvements were seen especially in the 6MWT and 5STS. Although preoperative home-based exercises did not have a consistent effect on rates of serious postoperative complications, they have the potential to shorten hospital stay and improve parameters such as pain and frailty.

Preoperative aerobic exercises, respiratory exercises, chest physiotherapy and lifestyle changes, help prepare patients physically and psychologically for surgery by improving lung capacity, strengthening respiratory muscles, enhancing cardiovascular endurance, and facilitating early postoperative mobilization. These approaches also reduce anxiety and increase motivation, leading to better postoperative compliance [23,24,25].

Studies reporting similar results to our review also emphasized that preoperative exercise is effective in improving respiratory function and physical capacity, and also reduces the LOS [24,26,27]. In a study examining the effectiveness of exercise programs applied before non-small cell lung cancer surgery, it was found that preoperative exercises significantly reduced the risk of postoperative pulmonary complications, shortened the duration of hospitalization, and increased exercise capacity, while emphasizing that exercise programs were quite safe [28].

In addition, a meta-analysis evaluating the effectiveness of respiratory exercises before lung cancer surgery showed that preoperative respiratory exercises significantly reduced the LOS (3.44 days shorter on average), pneumonia and postoperative pulmonary complications; 6MWT provided an increase of approximately 20.2 m. The combination respiratory exercises with aerobic exercises further reduced the length of hospital stay. No significant change was observed in respiratory functions and QoL [23].

Despite these benefits, patients often encounter difficulties in attending hospital-based programs due to transportation issues, time constraints, financial burden, physical discomfort and infection risks. Therefore, home-based programs offer intensive and regular interventions in places where patients feel more comfortable and, with the help of developing technology, can be effective alternatives to hospital-based programs. In a feasibility study evaluating the applicability of home-based preoperative exercise programs in patients undergoing lung cancer surgery, Machado et al. reported that these interventions were effective in increasing physical performance and preoperative endurance. In addition, it was highlighted that they could provide significant improvements in 6MWT and 5STS scores [6].

In a meta-analysis of 16 separate studies, when home-based pulmonary rehabilitation was compared with hospital-based standard care and its effects on functional exercise capacity and QoL were analyzed, it was similar to the hospital-based approach, achieving significant improvements in increasing exercise capacity and health-related QoL in Chronic Obstructive Pulmonary Disease (COPD) patients [29]. The observation that home-based exercise produced outcomes similar to those of supervised hospital-based interventions is noteworthy, indicating that remotely supervised home-based programs may serve as a feasible alternative for patients who cannot adhere to in-person clinical follow-up.

The ease of implementation of home-based programs may increase patient compliance and ensure the continuity of the programs. This is a significant advantage, especially for people with limited access to hospitals. In studies evaluating the feasibility and acceptability of home-based exercise programs using fitness equipment, telerehabilitation and exercise prescription before lung cancer surgery, the majority of participants successfully completed the program and demonstrated that the exercises were applicable [30,31]. In the studies included in our review, participants received high-quality interventions assessed with the i-CONTENT tool, were contacted by telephone when necessary, and the vast majority of participants completed the interventions. However, methodological differences and the variety of evaluation criteria between the studies limit the generalizability of the results. In addition, the lack of a clear standard for the duration and intensity of exercise protocols makes comparisons between trials difficult.

As seen in the results of our review, although preoperative home-based rehabilitation practices are effective approaches on parameters such as physical capacity, QoL and duration of hospitalization after surgery, compliance with and completion of home-based interventions are not always possible. Patient compliance, which affects participation in preoperative exercise programs in pulmonary surgeries, is also an important factor to be taken into consideration when planning and conducting studies [5,32]. In a prospective observational study investigating barriers to completing a home rehabilitation program prior to lung cancer surgery, 40% of participants failed to complete the program, with reasons including disease progression, advancing surgery date and lack of motivation [9]. While the age, functional capacity and motivation of the participants play a crucial role in treatment compliance, it shows that certain preoperative protocols can reduce postoperative complications [32]. These factors should be taken into account in future studies and clinics to improve the effectiveness of home-based interventions used before surgery.

All studies included in our review did not report the same significant improvements in respiratory parameters, postoperative complications, functional capacity or LOS [7,8,21]. This may be due to the fact that although studies in the literature show that preoperative home-based interventions can produce similar results to hospital-based approaches, remote monitoring of exercises and limited direct interaction with instructors may not be appropriate for patients of all ages, education levels and cultures, and the progressive nature of diseases that may require pulmonary surgery.

In addition, as stated in the study by Patel et al., other factors such as COPD (*OR*: 4.21), history of myocardial infarction (*OR*: 9.33), and thoracotomy (*OR*: 10.79) are also important reasons for prolonged hospital stays of more than five days [19]. In this context, digital health solutions such as telerehabilitation systems, mobile applications and wearable devices, as used in the studies of Patel et al. and Singer et al. developed in recent years, allow for closer monitoring of patients’ exercise processes and immediate intervention according to individual needs [33,34,35]. Such technology-supported models may help overcome some of the barriers associated with home-based training.

Regular monitoring of exercise programs and strengthening feedback mechanisms through synchronous or asynchronous monitoring systems are among the potential strategies that can increase the effectiveness of interventions [31,36,37]. Therefore, the technological support may further optimize the success of preoperative approaches.

The majority of the population included in our study consists of patients who will undergo lung cancer surgery. Recent important RCTs in cancer populations have demonstrated that structured preoperative exercise programs provide significant benefits not only during the preoperative phase but also during postoperative recovery and throughout adjuvant treatment [38]. Continuing hospital-based or home-based exercise after surgery has been reported to significantly improve functional capacity, reduce symptom burden, and improve long-term QoL outcomes in cancer patients. Although our review focused primarily on the preoperative phase, these findings suggest that continuing exercise programs at home after surgery may play an important role in improving long-term postoperative outcomes in lung cancer patients [38,39,40]. Therefore, integrating a postoperative exercise component into prehabilitation programs for patients undergoing future complex pulmonary surgery may be considered.

There are several limitations to our review. A total of 6 trials were included in our review, of which only 3 were RCTs. A meta-analysis could not be performed because there were differences between the measurement tools, rating scales and methodological approaches of the included studies. This prevents our study from being at the top of the evidence pyramid and is considered an important limitation of the study.

The long-term effects of home-based preoperative exercises have not been investigated comprehensively enough in the studies included in our review. In addition, we acknowledge the heterogeneity among the included studies, particularly the differences in patient populations such as lung transplant candidates versus resection surgery patients. However, given the shared clinical trajectory of preoperative pulmonary optimization and the growing use of home-based interventions across surgical contexts, we included both to provide a broader insight into feasibility and functional outcomes. As in the scoping review by Wade-Mcbane et al. (2023), which examined prehabilitation practices in the lung cancer pathway, and the meta-analysis by Pu et al. (2021), which evaluated breathing exercises before lung resection surgery, our review also adopted a descriptive synthesis approach rather than statistical pooling to avoid inappropriate generalization due to methodological diversity [11,23].

Postoperative complications, permanent effects on QoL, and changes in physical capacity are important topics to focus on in future research. Future research on home-based preoperative exercise programs should prioritize the development of standardized intervention frameworks for each surgical method and disease to minimize heterogeneity across studies. Consensus on key components such as exercise modality (aerobic, resistance, respiratory), training intensity, progression criteria, session frequency, and adherence and participation monitoring strategies will promote comparability and reproducibility across studies. Future research should also investigate the sustainability of behavioral changes achieved in the preoperative period, their continuation in the postoperative period, and the long-term consequences of these changes. Additionally, monitoring individual adherence to exercise programs and analyzing the factors that influence adherence could increase program success.

Integration of digital health technologies (mobile apps, wearable sensors, remote monitoring systems) can facilitate real-time adherence monitoring and improve intervention fidelity by providing objective training data. Finally, future high-quality multicenter RCTs with aligned protocols and coordinated outcome assessment schedules should be designed to establish a stronger and more generalizable evidence base for the role of home-based preoperative exercise programs.

## 5. Conclusions

This systematic review suggests that preoperative home-based exercise programs may offer promising benefits for patients undergoing pulmonary surgery, including improvements in functional capacity, respiratory function, QoL, and shorter hospital stays. These programs are cost-effective, accessible, and adaptable to individual needs. However, due to limited and heterogeneous evidence, strong conclusions cannot yet be drawn. Further high-quality RCTs with larger sample sizes are needed to confirm these preliminary findings and explore their long-term impact.

## Figures and Tables

**Table 1:** Characteristics of the Included Studies.

Authors, Year [Ref]	Country	Study Design	Participants n of, Definition	Age ± SD	Gender n Males (%)
Patel et al., 2023 [19]	Canada	RCT	95 lung cancer patients (45MFS, 50 CG)	MFS: 65.53 ± 8.66 CG: 68.78 ± 8.79	40 (%42.1)
Liu et al., 2020 [7]	China	RCT	73 lung cancer patients (37 PG, 36 CG)	HPG: 56.2 ± 10.3 CG: 56.2 ± 8.7	23 (%31.5)
Machado et al., 2024 [20]	Portugal	RCT	41 lung cancer patients (21 PHET, 20 CG)	PHET = 66.4 ± 7.2 CG = 68.7 ± 10.3	PHET = 13 (%65) CG = 15 (%71.4)
Saito et al., 2021 [21]	Japan	Retrospective Cohort Study	HBPPR = 51 NSCLC patients Non-HBPPR = 93 NSCLC patients	HBPPR = 73.0 ± 6.0 Non-HBPPR = 71.3 ± 7.3	HBPPR = 39 (%76.5) Non-HBPPR = 61 (%65.6)
Massier et al., 2020 [8]	Canada	Retrospective Cohort Study	159 lung transplant candidate	49.8 ± 14.0	91 (%57.2)
Singer et al., 2018 [22]	United States	A pilot study	15 lung transplant candidates	63 ± 5.7	10 (%66.6)

RCT: randomized controlled trial, MFS: move for surgery group; PG: prehabilitation group, CG: control group, HPG: home-based prehabilitaon group; PHET: preoperative home-based exercise training, HBPPR: home-based preoperative pulmonary rehabilitation, NSCLC: Non-Small Cell Lung Cancer.

**Table 2:** PedRO Quality Assesment.

Criteria	[20]	[19]	[21]	[7]	[8]	[22]
Eligibility criteria	Yes	Yes	Yes	Yes	Yes	Yes
Randomized allocation	Yes	Yes	No	Yes	No	No
Concealed allocation	Yes	Yes	No	Yes	No	No
Comparable at baseline	Yes	Yes	Yes	Yes	Yes	Yes
Blinded subjects	Yes	No	No	No	No	No
Blinded therapists	No	Yes	No	No	No	No
Blinded assessors	Yes	Yes	No	Yes	No	No
Adequate follow-up	Yes	Yes	Yes	Yes	Yes	Yes
Intention-to-treat analysis	Yes	Yes	Yes	No	Yes	Yes
Between-group comparisons	Yes	Yes	Yes	Yes	No	Yes
Point estimates and variability	Yes	Yes	Yes	Yes	Yes	Yes
Total Score	9/10	9/10	5/10	7/10	4/10	5/10

**Table 3:** Procedures and Main Findings of the Included Studies Investigating the Effects of Home-Based Preoperative Training.

Authors, Year [Ref]	Including Criteria	Interventions	Main Findings
Patel et al., 2023 [19]	Age: ≥18 years Clinical stage: I, II, or IIIa NSCLC Subjects: Individuals who are candidate for thoracic surgery and own a smartphone, tablet, or laptop.	MFS group received a Fitbit^®^ device. Education on aerobic exercise, deep breathing, healthy eating, sleep hygiene, and smoking cessation. Deep breathing: 10 breaths, 3 times/day. Daily steps: increase baseline average by 10% weekly (up to 10,000 steps/day). Control group: usual care (smoking cessation education only). All participants received standard postoperative management.	Hospital stay >5 days: 7% in MFS group vs. 24% in control group. Significant preoperative improvements in: Mobility (*p* = 0.008); Pain/discomfort (*p* < 0.001) MFS group showed significant improvement in overall EQ-5D-5L™ health score (*p* < 0.001), while the control group showed no change (*p* = 0.884). Daily step counts increased from baseline in the MFS group.
Liu et al., 2020 [7]	Age: <70 years newly Subjects: Patients with newly suspected or confirmed diagnosis, clinical stages I-III, and planned VATS lobectomy.	The HPG performed; Aerobic endurance exercise 30 min, ≥3 days/week (guided by Borg scale & heart rate). Resistance training with elastic bands: 10–12 reps × 3 sets. Respiratory exercises: at least 10 min, twice daily for 2 weeks. Relaxation training: imagery + music-assisted relaxation. Educational booklet provided. Weekly support phone calls. CG received the usual clinical care	6MWT increased by 60.9 m in the home-based prehabilitation group (*p* < 0.001). FVC improved by 0.35 L in the intervention group (*p* = 0.021). No significant between-group differences in 30-day postoperative complications (incidence or severity).
Machado et al., 2024 [20]	Age: ≥18 years Subjects: Patients with suspected or confirmed lung cancer (clinical stage IIIA or lower) scheduled for surgery Additional criteria: At least 2 weeks available before surgery, able to exercise, and voluntarily agreed to participate.	PHET includes; Aerobic training + resistance training combination. Weekly phone guidance. Duration: mean 3.6 ± 0.7 weeks. Outcomes assessed via EORTC-QLQ-C30, HGS, ISWT, and 5STS at baseline, post-intervention, and 4–5 weeks post-surgery.	Global QoL significantly higher in the PHET group after surgery (MD = 12.4; *p* = 0.029). PHET group showed: Better preoperative 5STS performance and better postoperative ISWT performance (*p* < 0.05). High adherence rates: 103% ± 19.8% (aerobic training) and 92.1% ± 33.1% (resistance training). No significant differences for other secondary outcomes.
Saito et al., 2021 [21]	Subjects: Patients with primary NSCLC scheduled for lung resection (lobectomy, sublobar resection, or wedge resection).	The HBPPR group received 4 exercises: abdominal crunch, chest stretching, abdominal breathing, and respiratory muscle training. Incentive spirometer (Coach2) used for respiratory muscle training. Program duration: 2–4 weeks. Both groups (post-op): early mobilization, breathing & coughing exercises, aerobic activity (walking or ergometer). Postoperative complications were assessed using the Clavien-Dindo classification and the length of hospital stay was monitored.	Home-based preoperative program reduced overall postoperative complications (*p* = 0.04). Significant reduction in Clavien-Dindo class I complications (RR = 0.55; 95% CI 0.30–1.02; *p* = 0.05). No significant differences in -LOS, intercostal catheter duration, and higher Clavien-Dindo complication classes.
Massierer et al., 2020 [8]	Age: ≥18 years Subjects: Individuals who underwent single or double lung transplantation at CHUM (Notre Dame Hospital), with available pre-transplant 6MWT data	Home-based prehabilitation: warm-up, aerobic exercise, strengthening, stretching, cool-down. Functional capacity assessed using 6MWT during evaluation, pre-transplant, and 1 month post-transplant.	Change in 6MWT pre-transplant: 25.8% improved (+85.8 m), 45.3% decreased (−109.8 m), and 28.9% showed no change. Moderate correlation between last pre-transplant and post-transplant 6MWT (r = 0.528; *p* < 0.001). Changes in pre-transplant 6MWT not associated with mechanical ventilation duration, total hospital LOS, and ICU LOS.
Singer et al., 2018 [22]	Subjects: English-speaking outpatients with home oxygen equipment capable of delivering prescribed supplemental oxygen and an SPPB frailty score ≤ 11	Aidcube™ mobile app intervention. Phase 1: In-person assessment (SPPB frailty score, grip strength, 6MWT) + personalized exercise plan. Phase 2: 8-week home exercise program logged through the app. Real-time monitoring allowed adjustments based on feedback. Focus on motivation, adherence, and personalized goal setting.	Frailty improved in: 54% of participants by SPPB; 62% of participants by FFP (*p* = 0.08 and *p* = 0.07, respectively; borderline significance). The majority of frail participants were no longer classified as frail after the intervention. No significant improvements in 6MWT, grip strength, and activity indices.

NSCLC: Non-Small Cell Lung Cancer; MFS: move for surgery group; CG: control group; EQ-5D-5L™: EuroQol-5 Dimension 5 Level; VATS: video assisted thoracic surgery; HPG: homebased prehabilitaon group; PHET: preoperative home-based exercise training; EORTC-QLQ-C30: European Organization for Research and Treatment of Cancer (EORTC) Quality of Life Questionnaire C30 (QLQ-C30); HGS: Handgrip strength; ISWT: Incremental Shuttle Walk Test; 5STS: Five-times sit-to-stand test; QoL: Quality of Life; ICU: Intensive Care Unit; LOS: Length of Stay, 6MWT: 6-min walk test; MD: Mean difference; CI: Confident interval; RR: Relative risk; SPPB: Short Physical Performance Battery; FFP: Fried Frailty Phenotype.

**Table 4:** Consensus on Therapeutic Exercise and Training (I-CONTENT) Tool Assessment.

	Machado et al. (2024)	Patel et al. (2023)	Saito et al. (2021)	Liu et al. (2020)	Massierer et al. (2020)	Singer et al. (2018)
1.	✓	✓	✓	✓	✓	✓
2.	✓	✓	✓	✓	✓	✓
3.	✓	✓	✓	✓	✓	✓
4.	N/A	N/A	N/A	N/A	N/A	N/A
5.	✓	✓	✓	✓	✓	✓
6.	✓	✓	✓	✓	✓	✓
7.	✓	✓	N/A	✓	N/A	✓

1. Patient Selection, 2. Dosage of the Exercise Program, 3. Type of the Exercise Program, 4. Qualified Supervisor, 5. Type and Timing of Outcome Assessment, 6. Safety of the Exercise Program, 7. Adherence to the Exercise Program.

## Data Availability

As this work is a systematic review, no original datasets were generated. Processed data supporting the findings are available from the authors upon reasonable request.

## Supplementary Materials

The following supporting information can be downloaded at: https://www.mdpi.com/article/10.3390/jcm14238605/s1, Table S1: PRISMA 2020 Abstracts Checklist; Table S2: PRISMA 2020 Checklist.

## Appendix A

Keywords for searching

In this systematic review, a comprehensive search strategy was developed to identify relevant studies. The keywords listed below were employed across multiple databases to ensure an exhaustive exploration of the literature. These terms were carefully selected to encompass a wide range of terminology related to pulmonary surgeries, lung-related procedures, and associated clinical interventions. By utilizing this search strategy, we aimed to capture all eligible studies addressing the research question and minimize the risk of missing pertinent data.

The following keywords were used:

“pulmonary surgery” OR “pulmonary surgeries” OR “pulmonary surgical” OR “pulmonary surgical procedure” OR “lung resection” OR “pulmonary resection” OR “lung cancer surgery” OR “lung transplantation” OR ”pulmonary lobectomy” OR “COPD Surgery” OR “segmentectomy” OR “bilobectomy” OR “pneumonectomy” OR “lung transplant recipients” OR “chronic obstructive pulmonary disease surgery” OR “lung volume surgery” OR “lung volume reduction” OR “lobectomy”, “VATS” OR “video-assested thoracic surgery” OR “pulmonary sleeve resection” OR “bronchial sleeve resection” OR “wedge resection”

AND

“unsupervised exercise” OR “home-based rehabilitation” OR “home-based physical therapy” OR “home-based exercise” OR “home-based training” OR “home-based exercise training” OR “home-based exercise therapy” OR “home-based pulmonary rehabilitation” OR “home-based respiratory therapy” OR “unsupervised pulmonary rehabilitation” OR “unsupervised exercise training” OR “unsupervised training” OR “unsupervised exercise therapy” OR “unsupervised exercise” OR “unsupervised physical therapy” OR “unsupervised respiratory therapy” OR “living environment rehabilitation” OR “living environment training” OR “living environment exercises” OR “living environment pulmonary rehabilitation” OR “home care rehabilitation” OR “home care training” OR “outpatient rehabilitation” OR “outpatient training” OR “outpatient exercises” OR “outpatient pulmonary rehabilitation”

AND

“Preoperative” OR “perioperative” OR “preoperational” OR “before surgery” OR “preoperative care” OR “preoperative rehabilitation” OR “before operation” OR “ prehabilitation”

SEARCH RESULTS

Pubmed

Search	Actions	Results
#4	Search: #1 AND #2 AND #3	68
#3	Search: “preoperative” OR “perioperative” OR “preoperational” OR “before surgery” OR “preoperative care” OR “preoperative rehabilitation” OR “before operation” OR “prehabilitation”	586,131
#2	Search: “unsupervised exercise” OR “home-based rehabilitation” OR “home-based physical therapy” OR “home-based exercise” OR “home-based training” OR “home-based exercise training” OR “home-based exercise therapy” OR “home-based pulmonary rehabilitation” OR “home-based respiratory therapy” OR “unsupervised pulmonary rehabilitation” OR “unsupervised exercise training” OR “unsupervised training” OR “unsupervised exercise therapy” OR “unsupervised exercise” OR “unsupervised physical therapy” OR “unsupervised respiratory therapy” OR “living environment rehabilitation” OR “living environment training” OR “living environment exercises” OR “living environment pulmonary rehabilitation” OR “home care rehabilitation” OR “home care training” OR “outpatient rehabilitation” OR “outpatient training” OR “outpatient exercises” OR “outpatient pulmonary rehabilitation”	42,866
#1	Search: “pulmonary surgery” OR “pulmonary surgeries” OR “pulmonary surgical” OR “pulmonary surgical procedure” OR “lung resection” OR “pulmonary resection” OR “lung cancer surgery” OR “lung transplantation” OR ”pulmonary lobectomy” OR “COPD Surgery” OR “segmentectomy” OR “bilobectomy” OR “pneumonectomy” OR “lung transplant recipients” OR “chronic obstructive pulmonary disease surgery” OR “lung volume surgery” OR “lung volume reduction” OR “lobectomy” OR “VATS” OR “video-assested thoracic surgery” OR “pulmonary sleeve resection” OR “bronchial sleeve resection” OR “wedge resection”	621,483

Scopus

Search	Actions	Results
4	#1 AND #2 AND #3	14
3	TITLE-ABS-KEY (“preoperative” OR “perioperative” OR “preoperational” OR “before surgery” OR “preoperative care” OR “preoperative rehabilitation” OR “before operation” OR “prehabilitation”)	714,115
2	TITLE-ABS-KEY (“unsupervised exercise” OR “home-based rehabilitation” OR “home-based physical therapy” OR “home-based exercise” OR “home-based training” OR “home-based exercise training” OR “home-based exercise therapy” OR “home-based pulmonary rehabilitation” OR “home-based respiratory therapy” OR “unsupervised pulmonary rehabilitation” OR “unsupervised exercise training” OR “unsupervised training” OR “unsupervised exercise therapy” OR “unsupervised exercise” OR “unsupervised physical therapy” OR “unsupervised respiratory therapy” OR “living environment rehabilitation” OR “living environment training” OR “living environment exercises” OR “living environment pulmonary rehabilitation” OR “home care rehabilitation” OR “home care training” OR “outpatient rehabilitation” OR “outpatient training” OR “outpatient exercises” OR “outpatient pulmonary rehabilitation”)	7554
1	TITLE-ABS-KEY (“pulmonary surgery” OR “pulmonary surgeries” OR “pulmonary surgical” OR “pulmonary surgical procedure” OR “lung resection” OR “pulmonary resection” OR “lung cancer surgery” OR “lung transplantation” OR “pulmonary lobectomy” OR “COPD Surgery” OR “segmentectomy” OR “bilobectomy” OR “pneumonectomy” OR “lung transplant recipients” OR “chronic obstructive pulmonary disease surgery” OR “lung volume surgery” OR “lung volume reduction” OR “lobectomy” OR “VATS” OR “video-assested thoracic surgery” OR “pulmonary sleeve resection” OR “bronchial sleeve resection” OR “wedge resection”)	153,880

Cochrane

ID	Search	Hits
#1	(“pulmonary surgery”):ti,ab,kw (Word variations have been searched)	132
#2	“pulmonary surgeries”	5
#3	“pulmonary surgical”	53
#4	“pulmonary surgical procedure”	0
#5	“lung resection”	975
#6	“pulmonary resection”	401
#7	“lung cancer surgery”	495
#8	“lung transplantation”	1605
#9	”pulmonary lobectomy”	190
#10	“COPD Surgery”	2
#11	“segmentectomy”	454
#12	“bilobectomy”	107
#13	“pneumonectomy”	1461
#14	“lung transplant recipients”	325
#15	“chronic obstructive pulmonary disease surgery”	1
#16	“lung volume surgery”	1
#17	“lung volume reduction”	332
#18	“lobectomy”	2246
#19	“VATS”	1205
#20	“video assisted thoracic surgery”	455
#21	“pulmonary sleeve resection”	2
#22	“bronchial sleeve resection”	3
#23	“wedge resection”	416
#24	#1 OR #2 OR #3 OR #4 OR #5 OR #6 OR #7 OR #8 OR #9 OR #10 OR #11 OR #12	4206
#25	#13 OR #14 OR #15 OR #16 OR #17 OR #18 OR #19	4377
#26	#21 OR #22	5
#27	#24 OR #25 OR #26	7687
#28	“unsupervised exercise”	212
#29	“home based rehabilitation”	484
#30	“home based physical therapy”	39
#31	“home based exercise”	1919
#32	“home based training”	378
#33	“home based exercise training”	203
#34	“home based exercise therapy”	54
#35	"home based pulmonary rehabilitation"	156
#36	“home based respiratory therapy”	1
#37	“unsupervised pulmonary rehabilitation”	1
#38	“unsupervised exercise training”	26
#39	“unsupervised training”	69
#40	“unsupervised exercise therapy”	6
#41	“unsupervised exercise”	212
#42	“unsupervised physical therapy”	0
#43	“unsupervised respiratory therapy”	0
#44	“living environment rehabilitation”	0
#45	“living environment training”	0
#46	“living environment exercises”	0
#47	“living environment pulmonary rehabilitation”	0
#48	“home care rehabilitation”	6
#49	“home care training”	8
#50	“outpatient rehabilitation”	706
#51	“outpatient training”	40
#52	“outpatient exercises”	0
#53	“outpatient pulmonary rehabilitation”	132
#54	#28 OR #29 OR #30 OR #31 OR #32 OR #33 OR #34 OR #35 OR #36 OR #37 OR #38 OR #39 OR #40 OR #41 OR #42 OR #43 OR #44 OR #45 OR #46 OR #47 OR #48 OR #49 OR #50 OR #51 OR #52 OR #53	3884
#55	“preoperative”	51,043
#56	“perioperative”	30,006
#57	“preoperational”	61
#58	“before surgery”	15,045
#59	“preoperative care”	6763
#60	“preoperative rehabilitation”	95
#61	“before operation”	3077
#62	“prehabilitation”	849
#63	#55 OR #56 OR #57 OR #58 OR #59 OR #60 OR #61 OR #62	84,050
#64	#27 AND #54 AND #63	14

Pedro

Search	Actions	Results
#1	Home based AND preoperative	40

Pubmed	68
Scopus	14
Cochrane	14
Pedro	40
Total	136
Duplicates	19
Unique Article	117
